# Trends in Canadian ophthalmology residency match outcomes

**DOI:** 10.36834/cmej.69809

**Published:** 2020-07-15

**Authors:** Jeffrey M. Mah, Irfan N. Kherani, Bernard Hurley

**Affiliations:** 1 Department of Ophthalmology, University of Ottawa, Ontario, Canada

## Abstract

**Background:**

To date, there exists no formal assessment of the competitiveness of the residency match for Canadian ophthalmology programs. The primary objective of this study was to use Canadian Resident Matching Service (CaRMS) data to describe trends in the number of positions, number of applicants and level of competition for the Canadian ophthalmology match.

**Methods:**

The number of positions and the number of applicants for each ophthalmology program were received from CaRMS for each cycle of the match from 2006-2017. The level of competition was calculated by dividing total number of applicants by the total number of positions in any given year.

**Results:**

The level of competition was consistently high with a median number of 2.0 applicants per anglophone Canadian Medical Graduate (CMG) position, 2.6 applicants per francophone CMG position and 32.5 applicants per International Medical Graduate (IMG) position. Over the study period, the level of competition decreased for francophone CMG and IMG positions and did not change for anglophone CMG positions.

**Conclusion:**

Consistently there are a greater number of applicants than positions for Canadian ophthalmology residency programs and therefore CMG applicants should be encouraged to apply to more than one discipline. The trends in the number of residency positions can be used to update supply projections for ophthalmologists and guide human resource planning.

## Abstract (fr)

**Contexte:**

À ce jour, il n’existe aucune évaluation officielle de la compétitivité du jumelage des résidents pour les programmes canadiens d’ophtalmologie. L’objectif principal de cette étude était d’utiliser les données du Service canadien de jumelage des résidents (CaRMS) pour décrire les tendances dans le nombre de postes, le nombre de candidats et le niveau de compétitivité pour le jumelage canadien en ophtalmologie.

**Méthodes:**

Le nombre de postes et le nombre de candidats pour chaque programme d’ophtalmologie ont été reçus du CaRMS pour chaque cycle du jumelage de 2006 à 2017. Le niveau de compétitivité a été calculé en divisant le nombre total de candidats par le nombre total de postes pour chaque année.

**Résultats:**

Le niveau de compétitivité était constamment élevé avec un nombre médian de 2,0 candidats par poste pour les diplômés canadiens en médecine (DCM) anglophones, 2,6 candidats par poste pour les DCM francophones et 32,5 candidats par poste pour les dipl00F4més internationaux en médecine (DIM). Au cours de la période de l’étude, le niveau de compétitivité a diminué pour les postes pour les DCM et DIM francophones et est demeuré inchangé pour les postes pour les DCM anglophones.

**Conclusions:**

On retrouve constamment un plus grand nombre de candidats que de postes pour les programmes canadiens de résidence en ophtalmologie et, par conséquent, les candidats DCM doivent être encouragés à postuler dans plus d’une discipline. Les tendances dans le nombre de postes de résidence peuvent être utilisées pour mettre à jour les prévisions de postes pour les ophtalmologistes et guider la planification des ressources humaines.

## Introduction

The demand for eye services in Canada is expected to increase significantly over the next decade. The most recent projections for Canada show that by 2030, the population ≥65 years old, the age demographic most affected by ophthalmic disease,^1-3^ will expand at four times the rate of that of ophthalmologists.^4^ This alarming trend has highlighted the need for human resource planning to ensure that the delivery of eye care is not compromised in the future. An important consideration in this planning is the number of ophthalmologists being trained in Canada as Canadian residency programs are the largest source of ophthalmologists entering the workforce.^5^ The trends in the number of residency positions available and the number of applicants to these positions are not well understood.

The Canadian Resident Matching Service (CaRMS) is the national, independent organization which administers the application, selection and matching services, collectively referred to as the residency match, for Canadian residency programs.^6^ CaRMS collects and houses unidentified data for each annual cycle of the residency match. This data can be used to understand trends in the match process and outcomes for a discipline.

We found no formal assessment of the residency match data for Canadian ophthalmology residency programs. The primary objective of this study was to describe trends in the ophthalmology residency match including number of available positions, number of applicants and level of competition. The secondary objective was to describe the outcomes for applicants who did not match to ophthalmology.

## Methods

### Study design

This is a retrospective review of the CaRMS data for ophthalmology residency matches from 2006-2017. The following data were received from CaRMS for each annual cycle of the residency match during the study period:

1. the number of positions available at each ophthalmology residency program,
2. the number of applicants to each ophthalmology residency program,
3. the number of individuals who applied to ≥1 ophthalmology residency program and were unmatched, and
4. the number of individuals who applied to ≥1 ophthalmology residency program and matched to an alternative discipline.

### Outcomes

The primary outcomes were the annual number of ophthalmology residency positions in Canada, the annual number of applicants to these positions and the annual level of competition for these positions. The number of distinct applicants to Canadian ophthalmology residency programs in any given year could not be directly obtained and therefore was estimated to be equal to the largest number of applicants to any one ophthalmology residency program in that year. The level of competition was calculated by dividing the total number of applicants by the total number of positions in any given year. Secondary outcomes included the percentage of applicants who did not match to ophthalmology who went unmatched and the percentage who matched to an alternative discipline.

CaRMS administers the residency match in two iterations.^7^ The first iteration is divided into two streams: a Canadian Medical Graduate (CMG) stream for graduates of Canadian and American medical schools, and an International Medical Graduate (IMG) stream for everyone else. The second iteration occurs for positions unfilled after the first iteration. Only data from the first iteration of the match were analyzed since during the study period there were only four residency positions unfilled after the first iteration. Additionally, this was felt to provide the most accurate reflection of the number of applicants and, by extension, the level of competition. Anglophone and francophone residency programs were also analyzed separately under the assumption that the applicants to each were largely distinct.

### Statistical analysis

Means and standard deviations were used to describe normally distributed variables and medians and ranges were used for variables with a non-normal distribution. Secular trends were evaluated over the study period using the Trend Test developed by Cuzick.^8^ The Trend Test performs the non-parametric test for trend across ordered groups which is an extension of the Wilcoxon rank-sum test. For variables with a non-linear distribution, Poisson regression was also used to describe the trend over time. A standard Poisson regression model was used since the likelihood ratio test–Vuong method for assessing overdispersion and zero-inflation did not indicate that an alternative model was preferred.^9^ The significance level was set at 0.05. All statistical analyses were performed using STATA version 12.

## Results

### Residency positions

**Figure 1:**
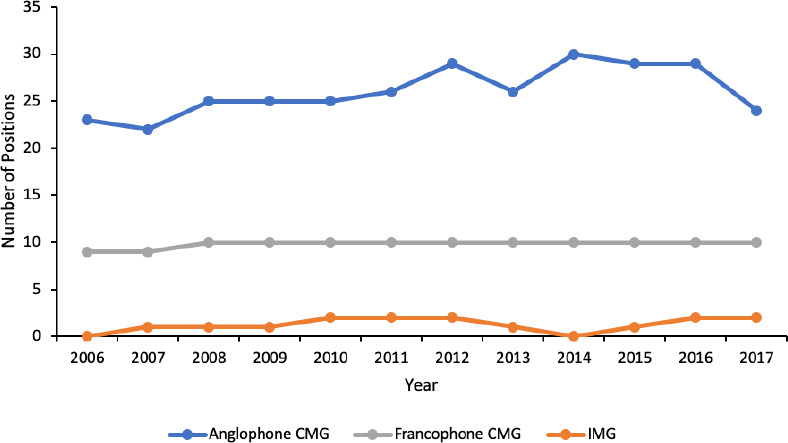
Annual number of ophthalmology residency positions for each applicant type in Canada.

The median annual number of ophthalmology residency positions was 25.5 (range 22-30) for anglophone CMGs, 10 (range 9-10) for francophone CMGs and 1 (range 0-2) for IMGs. The annual number of anglophone and francophone CMG positions increased over the study period (*p =* 0.04 and *p =* 0.03, respectively), while the number of IMG positions did not significantly change (Figure 1).

### Applicants

**Figure 2:**
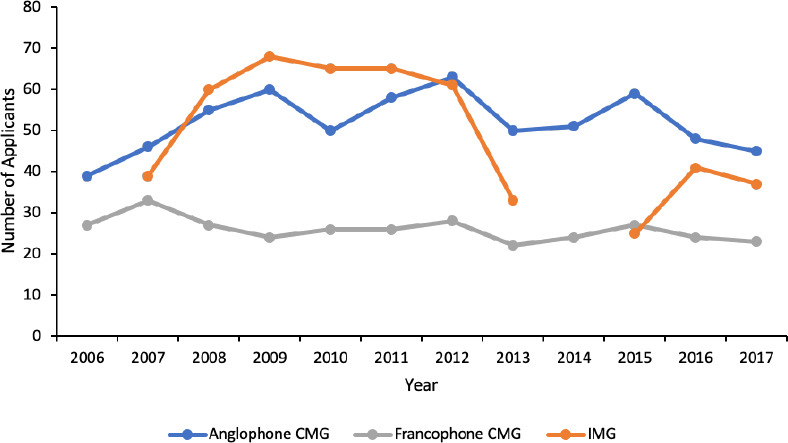
Annual number of ophthalmology applicants by applicant type in Canada. *Note: The number of IMG applicants were excluded in 2006 and 2014 as there were no IMG residency positions in these years*.

The median annual number of anglophone CMG applicants was 50.5 (range 39-63), francophone CMG applicants was 26 (range 22-33), and IMG applicants was 50.5 (range 25-68). There was no significant change in the number of anglophone CMG, francophone CMG or IMG applicants over the study period when evaluated using the Trend Test (Figure 2). Poisson regression, however, found that the number of IMG applicants decreased by 5% per year (IRR 0.95, 95% CI 0.92-0.97, *p* < 0.01).

### Level of competition

**Figure 3:**
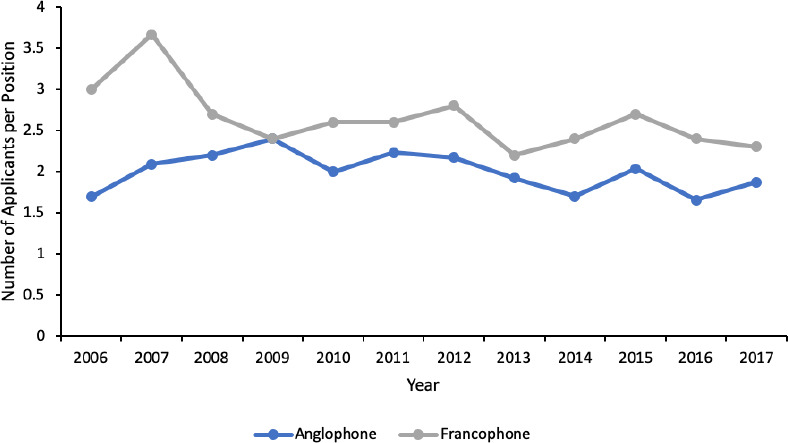
Annual level of competition for CMG ophthalmology residency positions by applicant type in Canada.

**Figure 4:**
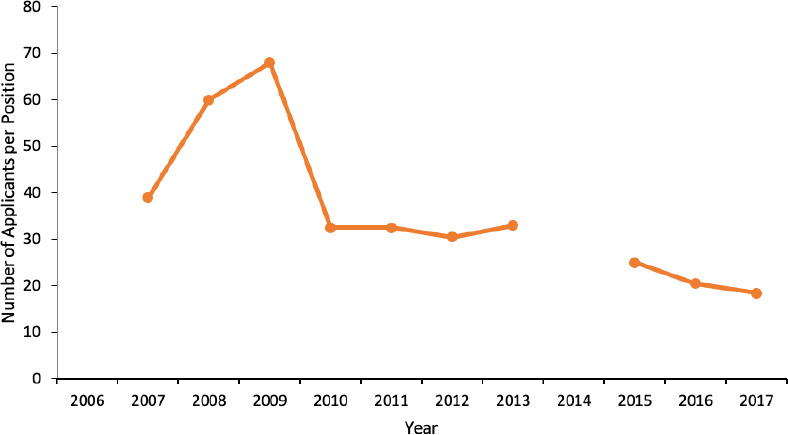
Annual level of competition for IMG ophthalmology residency positions in Canada. *Note: Level of competition could not be calculated in 2006 and 2014 as there were no IMG residency positions in these years*.

The level of competition was the highest for IMG applicants (median 32.5 applicants/position, range 18.5-68), followed by francophone CMG applicants (median 2.6 applicants/position, range 2.2-3.7) and the lowest for anglophone CMG applicants (median 2.0 applicants/position, range 1.7-2.4). The level of competition decreased for francophone CMG and IMG applicants (*p =* 0.03 and *p =* 0.01, respectively) and did not change for anglophone CMG applicants (Figures 3 and 4).

### Unmatched applicants

**Figure 5:**
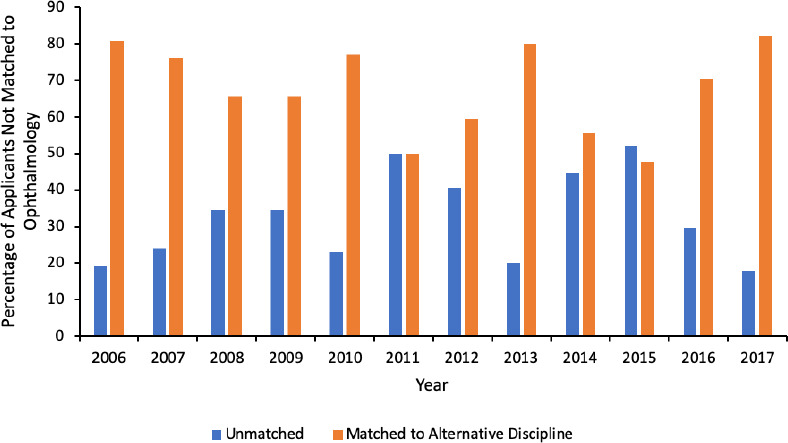
Annual outcomes of CMG applicants not matched to ophthalmology after the first iteration.

**Figure 6:**
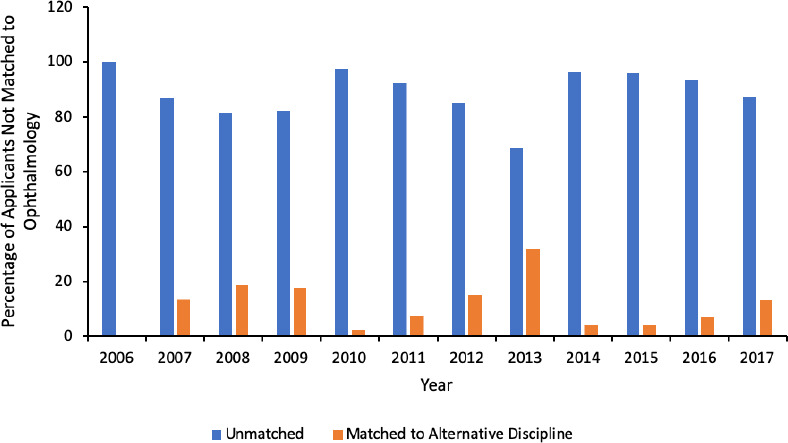
Annual outcomes of IMG applicants not matched to ophthalmology after the first iteration.

After the first iteration of the residency match, the majority of CMG applicants who did not match to ophthalmology matched to an alternative discipline (median 68%, range 48%-82%) compared to going unmatched (median 32%, range 18%-52%; Figure 5). On the contrary, the majority of IMG applicants who did not match to ophthalmology went unmatched after the first iteration (median 90%, range 68%-100%) compared to matching to an alternative discipline (median 10%, range 0%-32%, Figure 6).

## Discussion

Our study evaluated the residency match data for Canadian ophthalmology residency programs. We found that the level of competition for ophthalmology positions was consistently high with a median 2.0 applicants per anglophone CMG position, 2.6 applicants per francophone CMG position and 32.5 applicants per IMG position. When analyzing trends over the study period, we found that the level of competition decreased for francophone CMG positions secondary to an increase in the number of positions. For IMG positions, the level of competition also decreased secondary to a decreased number of applicants. For anglophone CMG positions, the level of competition was stable despite an increase in the number of positions.

These results can be used when counselling student applicants considering ophthalmology residency programs. In order to decrease the number of CMG applicants who go unmatched, the Association of Faculties of Medicine of Canada (AFMC) has advised counselling on parallel plans, i.e. applying to more than one discipline in the first iteration.^10^ Matching in the first iteration is preferred for CMG applicants as the number of available positions in the second iteration is unpredictable and, unlike in the first iteration where the majority of positions are reserved for CMG applicants, the positions in the second iteration are typically open to CMG and IMG applicants alike. Our results suggest that counselling for parallel plans is especially important for ophthalmology applicants as there are invariably more applicants than positions available, making it mathematically impossible for all applicants to successfully match. It appears that CMG applicants are already adopting this strategy, especially in recent years. About 70% of CMG applicants unmatched to ophthalmology matched to an alternative discipline in 2016 and 82% in 2017. Nevertheless, a continued emphasis should be placed on parallel planning in attempt to further reduce the number of unmatched CMG applicants.

While we found no previous studies evaluating ophthalmology match data from Canada, multiple studies have evaluated data from the United States.^11,12^ In concordance with our study, they found that it was competitive to secure an ophthalmology residency position with match rates of 72% between 2003 and 2008^12^ and 74% in 2011.^11^ They did not evaluate trends over time, however they were able to identify several variables predictive of matching including: USMLE scores, number of programs ranked, membership in the Alpha Omega Alpha medical honor society, and matriculation at a high ranking medical school (according to National Institutes of Health funding). The majority of these variables are not applicable to Canadian residency programs, with the exception of the number of programs ranked. Presumably, this variable acts as a surrogate for the number of interviews completed with stronger applicants being offered more interviews and having an increased likelihood of matching. To inform both applicants and residency programs alike, future studies are required to identify predictors of matching to Canadian programs.

Additionally, the results of our study can be used to inform human resource planning in the field of ophthalmology. The most recent study comprehensively evaluating the supply and demand of ophthalmologists in Canada was performed by Bellan in 2013.^4^ Supply was projected until 2030 using the Canadian Medical Association Physician Resource Evaluation Template,^13^ which relies on several variables including the annual number of Canadian residency program graduates. In their model, this number was set at 36 based on trend data from the Canadian Post-MD Education Registry and future estimates accounting for those currently in training or medical school. Our results suggest that this projection may underestimate the number of ophthalmologists entering the workforce from Canadian residency programs. Between 2013 and 2017, the number of positions in ophthalmology programs increased (median annual number of positions 40, range 36-41). While it remains unlikely that this change will allow the population of ophthalmologists to keep pace with the rapidly growing senior population in Canada, it does indicate a need for an updated projection of the supply of ophthalmologists in Canada.

The results of this study must be considered in the context of methodological limitations. Firstly, we were unable to directly obtain the total number of applicants to Canadian ophthalmology residency programs. Therefore, the highest number of applicants to any one program was used as a surrogate. This assumption could have underestimated the total number of applicants and by extension the level of competition. Secondly, the level of competition was calculated using the total number of individuals who applied to ophthalmology. This included individuals who may not have ranked ophthalmology as their first choice discipline, potentially overestimating the true level of competition.

## Conclusion

Consistently there are a greater number of applicants than positions for Canadian ophthalmology residency programs. CMG applicants to ophthalmology should be encouraged to parallel plan in order to decrease the rate of unmatched applicants. The trends in the number of residency positions available can be used to update supply projections for ophthalmologists and guide human resource planning.
